# Barriers and facilitators to faecal immunochemical testing in symptomatic populations: A rapid systematic scoping review and gap analysis

**DOI:** 10.1111/jep.14120

**Published:** 2024-09-18

**Authors:** Sienna Hamer‐Kiwacz, Hannah Berntsson, George Galloway, Ann‐Marie Tran, Jia Yun Tan, Daniel Hind, Matthew Kurien

**Affiliations:** ^1^ Division of Population Health, School of Medicine and Population Health The University of Sheffield Sheffield UK; ^2^ The Medical School The University of Sheffield Sheffield UK; ^3^ Division of Clinical Medicine, School of Medicine and Population Health The University of Sheffield Sheffield UK

**Keywords:** diagnosis, medical research, primary care, systematic reviews

## Abstract

**Aim:**

Faecal immunochemical testing (FIT) is used to triage people with signs or symptoms of a colorectal cancer (CRC). Recent guidelines have recommended further research to improve access, uptake and return of FIT. This systematic scoping review aims to understand the barriers and facilitators to FIT testing in symptomatic patients.

**Method:**

Qualitative, quantitative and mixed‐methods studies published after September 2013 were included. MEDLINE, EMBASE and PsycINFO databases were searched to identify publications examining barriers and facilitators to FIT. Initially, the data underwent thematic analysis, and subsequently, factors were aligned to components of the Capability, Opportunity, Motivation, Behaviour model. All outcomes are presented in adherence to the Preferred Reporting Items for Systematic Reviews and Meta‐Analyses guidelines.

**Results:**

One thousand two hundred thirty‐two papers were identified; 11 met the inclusion criteria. Barriers and facilitators were identified at the patient (e.g., knowledge), provider (e.g., general practitioner awareness) and service level (e.g., method of providing FIT kits). Factors were categorised into the subcomponents of the model: psychological capability (e.g., lack of FIT knowledge), reflective motivation (e.g., beliefs regarding FIT sampling and faeces being unhygienic) and automatic motivation (e.g., embarrassment, scary, anxiety provoking). Gaps in knowledge emerged in three domains: (1) patient experience, (2) FIT pathway and (3) healthcare professionals experience of FIT.

**Conclusion:**

This systematic scoping review provides a summary of the literature on FIT uptake, and identified factors across multiple levels and components. To increase adherence to FIT completion within primary care, a multifaceted theory and evidence‐based approach is needed to underpin future behavioural science interventions.

## WHAT DOES THIS PAPER ADD TO THE LITERATURE?

Interventions to improve faecal immunochemical testing (FIT) returns are needed; however, uncertainty exists about strategies that could maximise benefit. This study summarises evidence on barriers and facilitators to FIT, and maps findings to behavioural theory. This approach to enhancing FIT completion is novel and informs the design of future behavioural change interventions.

## 1 INTRODUCTION

Most colorectal cancers (CRC) are diagnosed in people with symptoms following presentation to primary care or direct to specialist.^1^ Having symptoms is recognised to increase an individual's pretest probability of having CRC and likelihood of having advanced disease. To improve prognosis and survival within this group at highest risk, there is a need to maximise efficiency in the diagnostic pathway, which could allow for earlier CRC detection. Detection of CRC within primary care poses significant challenges, as symptoms are often subtle, and may overlap with benign conditions.^2^, ^3^ This can lead to potential misinterpretation or delayed recognition. This can negatively impact survival, with recent modelling research demonstrating a survival disadvantage between 6.4% and 10.7% for a 2‐month diagnostic delay.^4^

In 2017, the National Institute for Health and Care Excellence (NICE) recommended faecal immunochemical testing (FIT) (DG30) to help triage patients with low‐risk symptoms of CRC in English primary care.^5^ Growing evidence supporting the superior diagnostic accuracy of FIT in comparison to symptoms, and taking into account real‐world experience and coronavirus disease 2019 (COVID‐19), contributed to FIT being more widely adopted in subsequent years.^6^, ^7^ This broadened indication for FIT was recently endorsed in national guidelines, ensuring expedited hospital investigations are now targeted at those having highest CRC risk.^8^, ^9^

Although FIT is now fully integrated within the lower gastrointestinal (LGI) faster diagnosis pathway, there is increasing evidence demonstrating variation in FIT returns within primary care.^10^, ^11^ This has led to a NICE recommending research that evaluates methods to improve access, uptake and return of FIT, particularly in certain under‐served groups.^9^ The focus on socio‐demographic factors is driven by a need to address health inequalities in cancer diagnosis and treatment. Identifying groups that are less likely to complete FIT could help to inform strategies, which could improve uptake and contribute to more equitable cancer outcomes. This aligns with national health priorities, such as the NHS England Core20PLUS5 initiative, which aims to reduce healthcare inequalities.^12^

Ensuring changes in FIT uptake and returns necessitates behavioural shifts, which is best guided by a theoretical understanding of the underlying behaviours.^13^ The Capability, Opportunity, Motivation, Behaviour (COM‐B) model is a behavioural theory that can provide insights into FIT testing behaviour.^14^ According to COM‐B, behaviour is the outcome of the interaction between three components: capability (psychological or physical abilities), opportunity (social or physical contexts) and motivation (automatic or reflective influences, such as emotions or beliefs and intentions). Positioned at the core of the Behaviour Change Wheel (BCW), COM‐B serves as the foundational element in supporting development of future interventions.^14^ The Theoretical Domains Framework (TDF) is a theoretical framework consisting of 14 domains, that aims to assist in the identification of determinants of behaviour and to assess implementation problems.^15^ Despite their demonstrated effectiveness in comprehending and shaping various health behaviours in a range of study designs,^16^, ^17^, ^18^, ^19^, ^20^ the application of this model to FIT testing has not previously been explored.

The aim of this rapid systematic scoping review was to identify differential return rates of FIT between different sociodemographic groups and characterise the literature on the barriers and facilitators to FIT testing within primary care in the context of the NHS in the UK and other OECD countries in symptomatic populations, map these onto the COM‐B model and the TDF, and identify gaps in the literature.^21^

The specific objectives were to

- Conduct systematic searches and selection of studies on the differential return rates of FIT between different sociodemographic groups and facilitators and barriers to FIT testing in the context of the NHS in the UK and OECD countries.
- Chart data from eligible studies.
- Present narrative and tabular summaries.
- Conduct a gaps analysis showing research priorities.

In addition to findings from primary qualitative studies, we sought to include insights and interpretations from the authors of quantitative studies as a form of ‘unconventional evidence’ that could provide useful information on barriers and facilitators to FIT testing.

## 2 METHODS

The review was registered at OSF.io and the protocol was published on ORDA, the University of Sheffield's data repository.^22^, ^23^ This review adheres to the Preferred Reporting Items for Systematic Reviews and Meta‐Analyses extension for Scoping Reviews (PRISMA‐ScR).^24^

### 2.1 Search strategy and study selection

We combined thesaurus and free text terms (Supporting Information S1) to search MEDLINE, EMBASE (via Ovid) and PsycINFO databases for papers published in English from September 2013 to September 2023. Although FIT was officially introduced into English primary care in 2017 as part of the NICE DG30 guidelines,^5^ we chose 2013 as our start date to potentially capture any preliminary research or pilot studies, which could have informed the implementation. Studies before September 2013 were excluded as older detection methods, like the guaiac‐based faecal occult blood test were the mainstay of diagnostic testing at this time. Electronic database searches were complemented by searching abstracts from meetings held by the American Society of Clinical Oncology and the European Society for Medical Oncology. We also hand‐searched the reference lists of identified full‐text articles. We also used the ‘find similar’ and ‘related articles’ features on Ovid and PubMed to identify other eligible citations.

Three review authors (Ann‐Marie Tran, George Galloway, Jia Yun Tan) independently screened titles and abstracts and potentially eligible full texts against the eligibility criteria. Disagreements were resolved through discussion with senior members of the review team (Daniel Hind, Hannah Berntsson, Sienna Hamer‐Kiwacz and Matthew Kurien).

Studies were considered eligible if they assessed barriers and facilitators to FIT uptake and returns in people with symptoms or signs suggestive of bowel cancer, and who were subsequently offered FIT as part of their diagnostic work‐up. Studies focusing on screening populations, including those who may have incidentally reported symptoms, were excluded. Eligible study designs included qualitative studies, mixed methods studies, surveys and quantitative studies of any kind. Only studies conducted in the UK or an Organisation for Economic Co‐operation and Development (OECD) country and published in English were included. A barrier was defined as a factor that hinders or obstructs FIT testing, while a facilitator was defined as a factor that supports or promotes testing.

Studies were excluded if they involved nonhuman participants or evaluated FIT uptake and returns in asymptomatic, screening populations. Additionally, studies conducted in countries with private healthcare systems and low‐income countries were excluded as their general practice settings are not comparable to that of the NHS in the UK or other OECD countries. Commentary or opinion publications without new data were also excluded.

### 2.2 Data extraction

The data extraction process was conducted independently by one reviewer (Jia Yun Tan) and subsequently verified by a second reviewer (George Galloway, Ann‐Marie Tran or Sienna Hamer‐Kiwacz) to ensure accuracy and consistency. A standardised framework was developed and employed to systematically capture various aspects of each study. Data extracted included study design, perspective (patient or healthcare professional), participant characteristics (e.g., age range, ethnic composition, gender and socioeconomic status) and research priorities.

### 2.3 Collating and summarising determinants of FIT return in behavioural terms

Due to the lack of formal qualitative data from more traditional qualitative research methods such as interviews, ‘unconventional qualitative material’, such as author interpretations and commentaries appearing in the included quantitative research articles, was also extracted. Three reviewers (GG, AMT and JYT) independently coded this material into the 14 domains of the TDF, within the three constructs of the COM‐B model using deductive thematic analysis.^13^, ^14^, ^25^ The material was coded using a predefined coding framework based on the constructs of the COM‐B model and the domains of the TDF. Coding was guided by the definitions of the TDF domains presented in Supporting Information S2. Dialogue between the coders resolved any discrepancies following the independent coding. Senior researchers MK, DH, SHK and HB oversaw unresolved differences through group discussion and reviewed and refined the themes to form a coherent pattern fitting the COM‐B model and TDF.

### 2.4 Gap analysis

This analysis followed an iterative process, wherein research gaps, priorities and recommendations were identified from the included studies, and members of the research team engaged in discussions regarding areas of weakness in both content and methodology within the reviewed body of research.

## 3 RESULTS

### 3.1 Study selection

**Figure 1:**
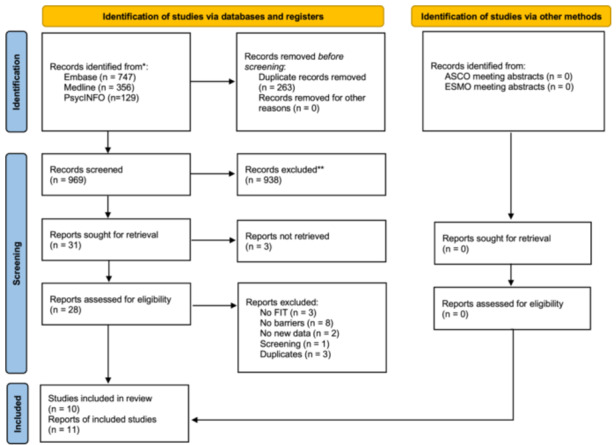
Preferred Reporting Items for Systematic Reviews and Meta‐Analyses flow chart. ASCO, American Society of Clinical Oncology; ESMO, European Society for Medical Oncology.

Excluding duplicates, 969 records were identified by the searches and screened (Figure 1). Thirty‐one papers met the inclusion criteria at the title/abstract stage, with three potentially relevant studies not retrievable. Of the remaining 28 studies, eleven papers met the inclusion criteria following full‐text review. All studies were derived from UK cohorts and were published between 2018 and 2023 (Table 1).

**Table 1:** Included study characteristics.

Study	Publication year	Study design	Perspective	Ethnic composition	Country	Healthcare setting
Bailey et al.^10^	2023	Quantitative	Patient	White, Asian, Black, mixed/other, unknown	UK	Secondary
Calanzani et al.^26^	2022	Qualitative	Patient	White British, white non‐British, other	UK	Primary
Coxon et al.^27^	2022	Quantitative	Patient	NR	UK	Secondary
Cripps et al.^28^	2023	Qualitative	Patient	NR	UK	Secondary
Delisle et al.^29^	2022	Qualitative	Patient	White or nonwhite	UK	Secondary
D'Souza et al.^30^	2021	Mixed methods	Patient	White, Asian, black, mixed, Chinese, not specified	UK	Secondary
Gil et al.^31^	2023	Qualitative	Patient	White British, other	UK	Primary
Ip et al.^32^	2022	Qualitative	Patient + GP	Patients: White, Black African/Caribbean, Indian, Asian/Asian BritishHealthcare Professional: English/Welsh/Scottish/Northern Irish/British/Other white background/Irish/Chinese/Mixed/multiple ethnic groups/Indian	UK	Secondary
Maclean et al.^33^	2023	Qualitative	Patient	NR	UK	Secondary
Von Wagner et al.^34^	2018	Qualitative	GP	NR	UK	Primary
Von Wagner et al.^35^	2019	Qualitative	GP	NR	UK	Primary

Abbreviation: GP, general practitioners; NR, not reported.

### 3.2 Study characteristics

Nine studies examined symptomatic patients' perspectives of FIT.^10^, ^26^, ^27^, ^28^, ^29^, ^30^, ^31^, ^32^, ^33^ Three studies considered perspectives from general practitioners (GPs).^32^, ^34^, ^35^ Studies were mainly qualitative evaluations (*n* = 8), with a mixed method study and two quantitative studies also included. All studies were conducted in the UK and study size ranged between 15 and 38,920 participants.^10^, ^32^ The setting for included studies included were either in primary (*n* = 4) or secondary care settings (*n* = 7, Table 1).

### 3.3 Differential rates of FIT return

Studies reported a lower likelihood of FIT returns amongst males.^10^, ^26^ In two studies, it was shown that younger patients were less likely to return FIT samples than older patients.^10^, ^27^ Across three studies, it was consistently observed that the most socioeconomically deprived patients had diminished FIT kit return rates.^10^, ^27^, ^28^ Individuals identifying as minority ethnicities (Asian, Black, mixed or other; nonwhite) and non‐English speakers also had a reduced likelihood of returning FIT kits.^10^, ^29^, ^34^ FIT returns were also identified in one study to be influenced by the time interval before bowel preparation for colonoscopy.^30^ This study involved symptomatic patients receiving FIT kits in the post, as opposed to receiving directly from their GP practice.

### 3.4 Identified barriers and facilitators to FIT

Seven studies contained unconventional qualitative material which provided insights into the barriers and facilitators of FIT.^28^, ^29^, ^30^, ^32^, ^33^, ^34^, ^35^ The majority of barriers that patients faced when returning FIT kits fell under the motivation section of the COM‐B model (Supporting Information S2). Examples from this category were that patients found FIT to be unhygienic or had feelings of disgust towards their stools,^29^ which was further categorised as reflective motivation under COM‐B, and the beliefs about consequences section of the TDF. Some patients also found that FIT was a scary, unpleasant or an anxious experience,^28^ which was classified as automatic motivation under COM‐B and emotion domain of the TDF. Embarrassment surrounding completing FIT tests^29^ can be categorised as either reflective motivation (social/professional identity) or automatic motivation (emotion), depending on the reasoning for this embarrassment.

Some of the research was categorised as psychological capability under COM‐B, an example of this was that patients found completing a FIT difficult, especially those aged 40–64,^33^ which was categorised as the knowledge domain under the TDF. Patients who had successfully used FIT previously were also more likely to use it again in the future^29^; this was also categorised as psychological capability and under the skills domain of the TDF.

Another factor that may cause barriers to people completing FIT is whether the patient experiences a language barrier or has low health literacy, is non‐English speaking, or is in a nonwhite population.^32^ These patients were less likely to engage with the information provided by their GPs and therefore less likely to return FIT samples.^34^ These factors were classified under the psychological capability section of COM‐B and the knowledge and skills domains of the TDF. One paper suggested that GP knowledge of the NICE guidelines for FIT can affect the number of kits given to patients^35^; this was categorised as psychological capability under COM‐B and knowledge under the TDF.

### 3.5 Gap analysis

The following gaps in knowledge emerged: (1) patient experience, (2) FIT pathway and (3) healthcare professionals FIT experience. Findings and recommendations for future research are described in Table 2.

**Table 2:** Gap analysis.

Field	Potential recommendations
Patient experience	Conduct qualitative studies, e.g., interviews, to gain a more in‐depth understanding of FIT perception, experience and satisfaction of using FIT, and willingness and intention to use and return FIT in different patients such as different age groups, ethnicities and social deprivations.^10^, ^29^, ^31^ Assess how patient preference for receiving FIT (postal, GP, central hub) could influence FIT return rates.^29^ Address barriers to incorrect use, either due to issues with comprehension or physical dexterity, by providing detailed and accessible instructions at primary care and secondary care levels, to minimise the need for test repetition.^29^
FIT pathway	Determine the optimal follow‐up of patients after nonreturn of FIT.^10^ Enhanced identification of high‐risk young patients.^10^ Address ethnic inequalities by implementing suitable support systems and counselling services and solve communication challenges, such as difficulties with hearing or vision impairment.^10^ Track and audit the use of FIT beyond the existing NICE referral guidelines to anticipate challenges from both the patient and the healthcare professional perspectives.^26^ Assess interventions, such as GPs texting normal results to patients, that ensure patients always receive their FIT result regardless of whether it is normal or abnormal.^31^ Evaluate measures to address shorter consultation times in deprived areas that negatively impact patient understanding of FIT.^31^
Healthcare professionals FIT experience	Conduct surveys to understand the attitudes of both primary and secondary care clinicians regarding the use of FIT,^33^ and to identify GP's actual and intended use of FIT in clinical practice.^35^ Conduct qualitative iterviews with healthcare professions to further understand the barriers and facilitators to implementing FIT as a rule‐out test for CRC in primary care.^34^, ^35^ Use discrete‐choice experiments to investigate whether test accuracy is the key factor in GP's decision to use FIT over the 2 week‐wait (2WW) referral.^34^ Assess whether the use of health literacy universal precautions by clinicians improves patient understanding of how to access and utilise FIT.^32^ Use longitudinal data to assess the relationship between awareness and actual use of FIT over time.^35^

Abbreviations: FIT, faecal immunochemical testing; GPs, general practitioners; NICE, National Institute for Health and Care Excellence.

## 4 DISCUSSION

This is the first scoping review to conduct a theoretical analysis of and generate insights into the barriers and facilitators to FIT in primary care. Research in this particular field is increasing; however, the existing body of work remains primarily descriptive up to this point. Our review has identified the socio‐demographic groups that tend not to return FIT tests, as well as a number of factors at patient, provider and service levels, which are barriers and facilitators to FIT testing. Our findings support younger patients, males, ethnic minorities and socioeconomically deprived groups being less adherent to FIT testing through the assessment of unconventional qualitative material. The qualitative material from the eligible articles identified could only be coded to two out of the three COM‐B model constructs, and four out of the 14 domains of TDF. The majority of these barriers were associated with ‘knowledge’ and ‘motivation’ within the COM‐B model. This suggests that much more work is needed to understand the determinants of FIT return in people with suspected bowel cancer, such as the symptomatic populations studied in this review.

Although the inclusion of unconventional qualitative material is increasing in healthcare services research,^36^, ^37^ using more rigorous methods of analysis, such as that suggested by the Joanna Briggs Institute (JBI)^38^ should be considered for further evidence synthesis. Furthermore, unconventional qualitative material may not have accurately represented patients' experiences, but rather was based on practitioners' perspectives. This emphasises the need for better primary qualitative research, which prioritises patients' voices, particularly those from underserved populations. We also did not undertake a formal critical appraisal of published studies, which could have informed understanding about the quality, reliability and validity of the synthesised evidence. However, these limitations are a result of the review's rapid nature and its clinical urgency, therefore we believe that these limitations have caused minimal impact on its trustworthiness, particularly so as many of the Cochrane's Rapid Reviews Method Group's recommendations for the conduct of rapid reviews were followed.^39^ Although our review provides a contemporary oversight of factors influencing FIT uptake, it is limited by a lack of randomised controlled and qualitative studies, all derived from the UK. This geographical bias limits generalisability and may overlook cultural barriers specific to other regions. Additionally, we failed to fully capture the cultural diversity within UK populations, where different communities may face distinct challenges or attitudes towards FIT. Future research should consider diverse cultural contexts both within the UK and internationally to ensure culturally sensitive interventions for improving FIT uptake.

Our study combined barriers and facilitators reported by patients and healthcare practitioners. While this approach provided a comprehensive overview of factors influencing FIT uptake, it also presents challenges in interpretation. Patient and practitioner perspectives may differ significantly, and combining them without clear distinction could potentially obscure important differences. For instance, what practitioners perceive as barriers might not align with patients' experiences, and vice versa. Furthermore, the weight given to each perspective in our analysis may not be balanced, potentially skewing our findings. Future research should aim to clearly delineate and compare patient and practitioner perspectives, allowing for a more nuanced understanding of how these different viewpoints interact and influence FIT uptake.

This review is timely and follows recent research recommendations by the NICE to evaluate methods to improve access, uptake and return of FIT. Across patient, provider and service levels we have identified multiple barriers and facilitators relating to psychological capability and motivation (reflective and automatic), such as patient knowledge of and their ability to complete FIT. Given the nature of the studies included (mostly cross‐sectional) and the lack of qualitative data, we cannot state which component represents the most important influence on FIT testing. To increase FIT testing, we would advocate focus on targeting all identified factors, specifically: (1) normalisation of gastrointestinal diseases, (2) communication, (3) FIT‐specific information and education for both patients and GPs and (4) methods of administering FIT testing. This proposed multifaceted theory and evidence‐based approach could help to underpin a novel behavioural science intervention, which targets improving FIT uptake, particularly in identified lower FIT uptake groups.

The merits of this approach are multiple and relevant to patients, clinicians and commissioners of colorectal services. Nonreturn of FIT samples can lead to CRC diagnostic delays, which adversely impacts treatments. Targeted strategies to improve FIT returns could enhance CRC case‐detection, which is line with Core20PLUS5, which aims to improve early cancer diagnosis (75% of cases diagnosed at stage 1 or 2 by 2028) and address health inequalities.^12^

Beyond the diagnostic delays is the consideration of costs. An FIT sample kit is estimated to cost £5.24.^40^ NHS England data highlights how 565,534 2‐week wait LGI referrals were made in 2022/23.^41^ Most referrals on this faster diagnosis pathway now require a FIT to be performed. If approximately 10% of patients do not return their FIT sample, then nearly £300,000 of FIT kits are estimated to be wasted per annum in England. While there is a direct cost associated with wasted test kits, the broader economic impact is less clear, as not all patients who fail to complete a FIT would have a positive result or a cancer diagnosis. Therefore, a more comprehensive health economic analysis is needed to fully understand the cost implications of nonreturned FIT tests, taking into account the probabilities of positive results, cancer diagnoses, and the potential costs and benefits of different follow‐up strategies for nonreturners.

## 5 CONCLUSION

Designing future behavioural change interventions targeting FIT returns, can be informed by the challenges in four areas: normalisation of gastrointestinal diseases, communication, FIT‐specific information and education and methods in providing FIT samples. This review also demonstrates a restriction or FIT uptake research to symptomatic UK populations, which should be accounted for when planning future studies.

## AUTHOR CONTRIBUTIONS

Matthew Kurien conceived the review. The review was designed by Sienna Hamer‐Kiwacz, Hannah Berntsson, Daniel Hind and Matthew Kurien. George Galloway, Jia Yun Tan, Ann‐Marie Tran ran searches and screened studies supported by Daniel Hind, Sienna Hamer‐Kiwacz and Hannah Berntsson. George Galloway, Jia Yun Tan, Ann‐Marie Tran, Sienna Hamer‐Kiwacz and Hannah Berntsson extracted data, supported by Daniel Hind, and Matthew Kurien. George Galloway, Jia Yun Tan, Ann‐Marie Tran and Daniel Hind undertook the analysis. Sienna Hamer‐Kiwacz, Hannah Berntsson, Daniel Hind, George Galloway, Jia Yun Tan, Ann‐Marie Tran and Matthew Kurien prepared the manuscript and all authors read, commented on and approved the final draft.

## CONFLICT OF INTEREST STATEMENT

The authors declare no conflicts of interest.

## Supporting information

Supporting information.

Supporting information.

Supporting information.

Supporting information.

## Data Availability

Data supporting the results reported can be found within the article.
