# Supplementary material for: Barriers and facilitators to faecal immunochemical testing in symptomatic populations: A rapid systematic scoping review and gap analysis
Source: J Eval Clin Pract. 2024 Sep 18;31(2):e14120. doi: 10.1111/jep.14120 (PMC11938400; doi:10.1111/jep.14120)
Supplement: Supplementary file 1 — Supporting information. [file JEP-31-0-s004.docx]

**COLORECTAL DISEASE: Authorship declaration**

Title of article: Barriers and facilitators to faecal immunochemical testing (FIT) in symptomatic populations: a rapid systematic scoping review and gap analysis

**PLEASE READ FIRST:**

The journal follows the recommendations of the International Committee of Medical Journal Editors (ICMJE) for manuscripts submitted to biomedical journals. According to these, authorship should be based on the following four criteria:

1. Substantial contributions to the conception or design of the work; or the acquisition, analysis, or interpretation of data for the work; AND

2. Drafting the work or revising it critically for important intellectual content; AND

3. Final approval of the version to be published; AND

4. Agreement to be accountable for all aspects of the work in ensuring that questions related to the accuracy or integrity of any part of the work are appropriately investigated and resolved.

All other contributors to the paper should be credited in the 'Acknowledgements' section.

I, the designated corresponding author of the above article submitted for consideration of publication to Colorectal Disease, hereby confirm that:

(i) all named authors agree to the submission of the paper to Colorectal Disease;

(ii) all authors who qualify for authorship under the criteria listed above have been named on the paper;

(iii) the paper is not currently under review by another journal; and

(iv) the paper has not been accepted for publication elsewhere.

Name of corresponding author: Dr Matthew Kurien

Signature of corresponding author:
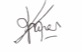


Date: 09/02/2024
