# Supplementary material for: Barriers and facilitators to faecal immunochemical testing in symptomatic populations: A rapid systematic scoping review and gap analysis
Source: J Eval Clin Pract. 2024 Sep 18;31(2):e14120. doi: 10.1111/jep.14120 (PMC11938400; doi:10.1111/jep.14120)
Supplement: Supplementary file 3 — Supporting information. [file JEP-31-0-s001.docx]

**Supplementary File 1**

[Ovid MEDLINE(R) and Epub Ahead of Print, In-Process, In-Data-Review & Other Non-Indexed Citations and Daily 1946 to September 14, 2023](https://ovidsp-dc1-ovid-com.sheffield.idm.oclc.org/ovid-new-b/ovidweb.cgi?&S=GKANFPEAHAACMPCIKPKJPEMIJBJCAA00&Database+Field+Guide=32)

1. Exp Colorectal Neoplasms/di [Diagnosis]
2. Occult Blood/
3. Faecal immunochemical test*.ti,ab,kw.
4. Fecal immunochemical test*.ti,ab,kw.
5. feces/ch
6. or/1-5
7. Symptomatic.mp.
8. barrier*.mp.
9. attitude*.mp.
10. Feasibility.mp.
11. Usability.mp.
12. perception*.mp.
13. experience*.mp.
14. engage*.mp.
15. prefer*.mp.
16. tolera*.mp.
17. willing*.mp.
18. Exp Qualitative Research/
19. Qualitative.ti,ab.
20. Themes.ti,ab.
21. or/7-20
22. 6 and 7 and 21
23. Limit 22 to (English language and yr=”2013-current”)
