# Supplementary material for: Barriers and facilitators to faecal immunochemical testing in symptomatic populations: A rapid systematic scoping review and gap analysis
Source: J Eval Clin Pract. 2024 Sep 18;31(2):e14120. doi: 10.1111/jep.14120 (PMC11938400; doi:10.1111/jep.14120)
Supplement: Supplementary file 4 — Supporting information. [file JEP-31-0-s003.docx]

**Supplementary File 2**

Capability Opportunity Motivation - Behaviour Model (COM-B) and Theoretical Domains Framework (TDF)

| **COM-B Component** |  | **TDF Domain** | **Definition** |
| --- | --- | --- | --- |
| Capability | Psychological | Knowledge | An awareness of the existence of something. |
|  |  | Skills | A learned ability to perform behaviours. |
|  |  | Memory, Attention and Decision Processes | The ability to retain information, keep focus and make a choice between two or more alternatives. |
|  |  | Behavioural Regulation | A behaviour aiming to manage or change observed actions. |
|  | Physical | Skills | A learned ability to perform behaviours. |
| Opportunity | Social | Social Influences | Interpersonal processes that can changes thoughts, feelings, or behaviours. |
|  | Physical | Environmental Context and Resources | Circumstances of a person's situation or environment that influences behaviour. |
| Motivation | Reflective | Social/Professional Role and Identity | A set of behaviours and personal qualities displayed in a social or work setting. |
|  |  | Beliefs about Capabilities | An acceptance of personal ability to perform behaviours. |
|  |  | Optimism | Confidence that desired goals will be achieved. |
|  |  | Beliefs about Consequences | Acceptance of the reality about outcomes of a behaviour in a given situation. |
|  |  | Intentions | A conscious decision to perform a behaviour. |
|  |  | Goals | Mental representations of outcomes or end states that an individual wants to achieve. |
|  | Automatic | Social Professional Role and Identity | A set of behaviours and personal qualities displayed in a social or work setting. |
|  |  | Optimism | Confidence that desired goals will be achieved. |
|  |  | Reinforcement | A dependent relationship between a given stimulus and a response. |
|  |  | Emotion | A individual’s experiential, behavioural, and physiological reaction to an event. |
